# Relationship between orthostatic hypotension and recurrence of benign paroxysmal positional vertigo

**DOI:** 10.1038/s41598-022-15029-5

**Published:** 2022-06-23

**Authors:** Moon Jung Kim, Gu il Rhim

**Affiliations:** 1 Department of Laboratory Medicine, Myongji Hospital, Hanyang University Medical Center, Goyang, South Korea; 2 One Otorhinolaryngology Clinic, 2 sicheong-ro, Paju, 10924 South Korea

**Keywords:** Neurology, Peripheral neuropathies

## Abstract

Blood pressure is maintained by a combined mechanism of the baroreceptor reflex and the vestibulosympathetic reflex. This study is intended to verify the hypothesis that the orthostatic hypotension (OH) seen when benign paroxysmal positional vertigo (BPPV) occurred may act as a factor that affects the recurrence of BPPV. The subjects of present study were selected from among 239 patients diagnosed with idiopathic BPPV. The average age of the group with OH was 59.3 years, and the age of the group without OH was 50.3 years, with a statistically significant difference (P = 0.013). It was shown that drug-taking increased the risk of OH occurrence by 4.08 times (C.I for exp(B): 1.20–13.77) compared to the group that did not take drugs. It was shown that the risk of recurrence of BPPV was significantly reduced in the no recurrence group compared to the multiple recurrence group when there was no OH (p = 0.000; aOR 0.0000002). Also, the risk of recurrence was significantly reduced in the no recurrence group compared to the multiple recurrence group when there was no drug-taking (p = 0.000 aOR 0.0000001). This study is the first study that studied the effect of OH on the recurrence of BPPV and showed the possibility that OH could partially influence the recurrence of BPPV.

## Introduction

The symptoms of vertigo can occur when there is an abnormality in any part of the visual system, the vestibular sensory system, and the somatosensory system for body balance, and the central nervous system that integrates the foregoing. Benign paroxysmal positional vertigo (BPPV) is one of the diseases that cause vertigo due to changes in the position of the vestibular sensory system, and the drop out of the otolith in the semicircular canals or excessive movements of the cupula cristae ampullaris is thought to be the cause of BPPV.

BPPV is also a disease that is prone to recurrence. In general, its recurrence rates within 1 year and 3 years are known to be 17% and 50%, respectively. The causes of recurrence are known to be age, vitamin D deficiency, chronic diseases such as hypertension and diabetes, and otologic diseases^1^.

The vestibular sensory system is known to send signals through the sympathetic nervous system to the nucleus tractus solitarius (NTS) and the rostral ventrolateral medulla (RVLM) maintain blood pressure in the event of position changes^2^.

The drop in blood pressure (BP) when standing up reduces the blood flow going to the inner ear subsequently causing ischemic excitation of the peripheral vestibular hair cells, which leads to the excitation of central vestibular nuclei thereby causing vertigo after hypotension. In addition, the excitation of the vestibular nucleus following the hypotension induces vestibulosympathetic reflex (VSR), and the reflex regulates blood pressure with a dual control (neurogenic and humoral control) mechanism^3^.

Diagnosis of hemodynamic orthostatic dizziness/vertigo requires: (A) five or more episodes of dizziness, unsteadiness or vertigo triggered by arising or present during upright position, which subsides by sitting or lying down; (B) orthostatic hypotension, postural tachycardia syndrome or syncope documented on standing or during head-up tilt test (HUTT); and (C) not better accounted for by another disease or disorder^4^.

Orthostatic hypotension (OH) is a disease of orthostatic vertigo, refers to cases where the systolic blood pressure drops by at least 20 mmHg, or the diastolic blood pressure drops by at least 10 mmHg within 3 min of standing up after lying down, and is diagnosed by measuring blood pressure^5^.

It is known that repeated occurrence of OH may result in hypoperfusion of the brain, which is responsible for the central nervous system, and cause blood flow insufficiency in the brainstem and the inner ear^6,7^.

Recent studies have shown that BPPV-induced otolith organ hypofunction can cause transient OH^8,9^. In this study, how OH and BPPV are associated with each other will be examined.

Since hypertension, diabetes, drugs, age, etc., known as the causes of OH are also known as risk factors for BPPV, this study is intended to verify the hypothesis that the OH seen when BPPV occurred may act as a marker of otolith organ hypofunction and thereby becoming a factor that affects the recurrence of BPPV.

## Methods

### Study subjects

This retrospective study was conducted with patients diagnosed with BPPV among patients who visited the One Otorhinolaryngology Clinic due to a symptom of vertigo from September 2020 to August 2021. When a patient who visited the hospital due to a symptom of vertigo showed any abnormal finding in the medical history taking, general otolaryngological examination, and physical examination using frentzel glass, the following tests were carried out immediately.

All patients did not intake alcohol within 12 h of the test, and drugs such as high blood pressure and diabetes, excluding stabilizers, were taken as usual.

After resting for at 15 min in a quiet room, the patient was laid down on the test table.

As for the sequence of the test, the blood pressure and pulse rate were measured twice at 1-min interval after resting for 3 min in a lying position, the table was raised to 70° for 30 s thereafter, the blood pressure and pulse rate were measured three times at 1-min intervals, and the patient was diagnosed with OH in case where the systolic blood pressure dropped by at least 20 mmHg or the diastolic blood pressure dropped by at least 10 mmHg. In cases where the patient felt severe vertigo during the test, the test was immediately stopped. In addition, all patients were subjected to a positional test and a positioning test using videonystagmography test. Posterior canal BPPV was diagnosed by observation of torsional upbeating nystagmus following Dix-Hallpike test. Horizontal nystagmus induced by supine roll test was considered as diagnostic sigh for lateral canal BPPV. Anterior canal BPPV is diagnosed when Dix-Hallpike positioning produces a down-beating nystagmus with torsional component. After diagnosis with otolithiasis, repositioning maneuvers were performed twice a week, and treatment was terminated when symptoms disappeared and the nystagmus was resolved based on video frentzel glass examination. All patients were diagnosed and treated by a physician.

Recurrence was diagnosed with symptoms that occurred one month after the end of the treatment and nystagmus confirmed with video frentzel glass, and treatment was carried out again.

### Patients data and variables

**Figure 1:**
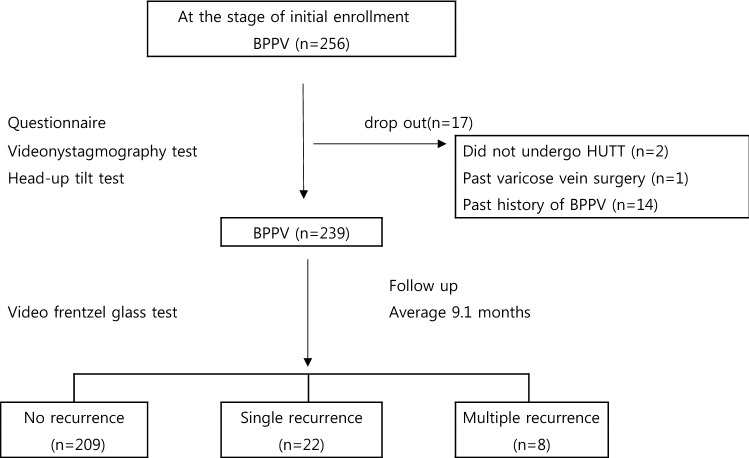
Flowchart of study design.

During the study period, there were 256 patients with idiopathic BPPV. Of them, two patients were unable to undergo HUTT, one patient was excluded due to past varicose vein surgery, and 14 patients were excluded due to a past history of BPPV. Finally, 239 patients were studied, and the average follow-up period was 9.1 months (Fig. 1). Through the patients’ medical records and questionnaire, age, sex, the location of otolith at the first occurrence of BPPV, otologic disease, hypertension, diabetes, OH, and whether drugs were taken (heart disease, cerebrovascular disease, psychiatric disease, thyroid disease, prostate disease) were identified. All patients gave their informed consent prior to their inclusion in the study and this study have been performed in accordance with the ethical standards in the 1964 Declaration of Helsinki and its later amendments. This study was approved by the Myongji Hospital Institutional Review Board.

### Statistical analysis

First, attempts were made to identify variables that cause statistical interactions as effect modifiers between OH and BPPV recurrence among the age, sex, otologic disease, the location of otolith, hypertension, diabetes, and drug-taking (heart disease, psychiatric disease, thyroid disease, prostate disease), which are variables investigated in the study.

Through Breslow-Day's test for homogeneity, age, drug use, hypertension, and diabetes passed the homogeneity test. In the tests of conditional independence, the Mantel–Haenszel test value was p = 0.963. Based on this, a model suitable for the study was designed. First, binary logistic regression analysis was performed to identify factors affecting OH among the study subjects, and polychotomous logistic regression analysis was used to study the effects of OH on BPPV recurrence. The fit of the model was p = 0.006, and polychotomous logistic regression analysis was carried out in the ascending method based on the multiple recurrence group among no recurrence group, single recurrence group, and multiple recurrence group. The model verified main effects, and finally, the overall statistical difference for the variables in the polynomial logistic regression analysis was verified through the Wald test using covariance. Statistical analyses were conducted using IBM SPSS ver. 19.0 (IBM Corp., Armonk, IL, USA). Statistical significance was considered to exist when p < 0.05.

## Results

As shown in Table 1, the prevalence of OH in the study subjects was 8.3% (n = 20). The average age of the group with OH was 59.3 years, and the age of the group without OH was 50.3 years, with a statistically significant difference (P = 0.013).

**Table 1:** Variables on orthostatic hypotension using binary logistic regression analysis.

Variables			Orhostatic hypotension		B	P value	Exp(B)	95% C.I.for EXP(B)	
			+ N = 20	− N = 219				Lower	Upper
Age(mean ± SD)			59.3 ± 11.8	50.3 ± 16.4	0.049	**0.013**	**1.050**	**1.011**	**1.091**
Sex	Male		6	59	− 0.217	0.695	0.805	0.272	2.382
	Female		14	160					
Hypertension	+	−	7	64	-0.983	0.146	0.374	0.099	1.409
			13	155					
Diabetics	+	−	6	29	1.073	0.108	2.925	0.791	10.819
			14	190					
Medications^a^	+	−	5	19	1.406	**0.023**	**4.081**	**1.209**	**13.774**
			15	200					

*SD* standard deviation, *B* unstandardized coefficient, *Exp(B)* the exponentiation of the B coefficient, *CI* confidence interval.

^a^The diseases of the drug-taking were arrhythmia, heart disease, antidepressant, thyroid disease, hypnotic and prostate disease.

Bold values indicate statistical significance (p < 0.05).

Drug-taking also had a significant effect on OH. It was shown that drug-taking increased the risk of OH occurrence by 4.08 times (P = 0.023; C.I for exp(B): 1.20–13.77) compared to the group that did not take drugs.

Sex, hypertension, and diabetes did not have any significant effect on the occurrence of OH in this study.

The characteristics of patients in each group are shown in Table 2. Of the entire 239 patients in Table 2, 209 (87%) were in the no recurrence group, 22 (9%) were in the single recurrence group, and 8 (3%) were in the multiple recurrence group, which comprised 7 patients with 2 recurrences and 1 patient with 3 recurrences. The locations of otoliths among the entire patients consisted of the posterior canal in 35 patients (15%), the lateral semicircular canal in 193 patients (80%), and multiple semicircular canals in 11 patients (5%). The male: female ratio was 1:2.7 and the ages of the patients were younger than 60 years in 155 patients (65%) and at least 60 years in 84 patients (35%) over 60 years old. Of the total patients, 71 (30%) had hypertension, 35 (15%) had diabetes, 20 (8%) had OH, and 24 (10%) were taking drugs. The diseases of the drug-taking patients were depression in 7 patients, thyroid disease in 5 patients, heart disease in 4 patients, arrhythmia in 3 patients, sleeping pill-taking in 3 patients, and prostate disease in 2 patients.

**Table 2:** Characteristics of the no recurrence, single recurrence, and multiple recurrence groups in BPPV.

BPPV recurrence episode	Canal location			Sex		Age		Hypertension		Diabetics		Orthostatic hypotension		Medications^b^	
	Post	Lat	Both	Male	Female	< 60	> 60	−	+	−	+	−	+	−	+
No n = 209	29	172	8	64	145	136	73	147	62	179	30	190	19	189	20
Single n = 22	4	16	5	0	22	14	8	15	7	17	5	21	1	18	4
Multiple^a^n = 8	2	5	1	1	7	5	3	6	2	8	0	8	0	8	0
Total n = 239(%)	35 (15)	193 (80)	11 (5)	65 (27)	174 (73)	155 (65)	84 (35)	168 (70)	71 (30)	204 (85)	35 (15)	219 (92)	20 (8)	215 (90)	24 (10)

*BPPV* Benign paroxysmal positional vertigo, *Post* posterior canal, *lat* lateral canal, *both* involves multiple canal.

^a^Includes seven people who recurred twice and one person who recurred three times.

^b^The diseases of the drug-taking were arrhythmia, heart disease, antidepressant, thyroid disease, hypnotic and prostate disease.

As shown in Table 3, in comparison using polychotomous logistic regression analysis with the no recurrence group using the multiple recurrence group as a control group, it was shown that the risk of recurrence of BPPV was significantly reduced in the no recurrence group compared to the multiple recurrence group when there was no OH (p = 0.000; aOR 0.0000002). Also, the risk of recurrence was significantly reduced in the no recurrence group compared to the multiple recurrence group when there was no drug-taking (p = 0.000; aOR 0.0000001). However, the absence of OH and drug-taking did not show any significant reduction in the risk of recurrence in the single recurrence group compared to the multiple recurrence group.

**Table 3:** Comparison of adjusted risks of BPPV recurrence among no recurrence, single recurrence, and multiple recurrence groups.

Risk factors	Categories or units	No recurrence vs multiple recurrence			Single recurrence vs multiple recurrence			P _wald_ (df)
		B	Sig	aOR (95% CI)	B	Sig	aOR (95% CI)	
Age	< 60	0.18	0.837	1.19 (0.21–6.78)	0.26	0.797	1.30 (0.17–9.74)	0.888
Sex	Male	1.15	0.294	3.15 (0.36–27)	− 15.35	0.990	2.14E−7	0.989
Hypertension	No	0.18	0.856	1.19 (0.17–8.33)	0.12	0.918	1.12 (0.11–11.24)	0.932
Diabetics	No	− 16.16	0.995	9.54E−8	− 16.81	0.995	4.98E−8	0.740
Orthostatic hypotension	No	− 15.71	**0.000**	**1.50E−7 (1.77E−8 to 1.27E−6)**	− 14.86		3.48E−7	0.439
Medications^a^	No	− 15.75	**0.000**	**1.44E−7 (4.07E−8 to 5.12E−7)**	− 16.72		5.47E−8	0.132

*BPPV* Benign paroxysmal positional vertigo, *B* unstandardized coefficient. *Sig.* significance probability, *aOR* adjusted odds ratio, *CI* confidence interval, *df* degrees of freedom.

^a^The diseases of the drug-taking were arrhythmia, heart disease, antidepressant, thyroid disease, hypnotic and prostate disease.

p values were calculated using polychotomous logistic regression analysis or Wald test.

Bold values indicate statistical significance (p < 0.05).

In addition, age, sex, hypertension, and diabetes did not show any significant difference in the risk of recurrence in both the no recurrence group and the single recurrence group compared to the multiple recurrence group. In conclusion, age, sex, hypertension, diabetes, OH, and drug-taking did not make any significant difference in the risk of recurrence among the three groups, and no statistically significant risk was observed.

## Discussion

Blood pressure is maintained by a combined mechanism of the baroreceptor reflex and the vestibulosympathetic reflex. The baroreceptor reflex of the carotid sinus is transmitted to the NTS through the glossopharyngeal nerve, and the baroreceptor reflex of the aortic arch is transmitted to the NTS through the vagus nerve^2^. Stimulation of the vestibular system according to posture changes lead to the excitation of the otolith organ and the relevant signals are transmitted to the NTS and the RVLM via the vestibular nucleus complex through the sympathetic nervous system. The information integrated in RVLM functions to maintain blood pressure.

According to the guidelines of the Bárány society, OH is defined by a significant reduction in systolic (> 20 mmHg) and/or diastolic (> 10 mmHg) blood pressure within 3 min upon standing from sitting or during head-up tilt test. It may cause orthostatic dizziness/vertigo or not. Although the most common cause of orthostatic dizziness/vertigo is probably OH, it is not the only cause. Thus, the nomenclature, orthostatic dizziness/vertigo and OH, should be used distinctively. Orthostatic dizziness/vertigo is a symptom while OH is a disorder^4^.

A large population-based study on OH during postural testing in adults aged > 20 years showed an overall OH prevalence of 4.8–9% but increased to 10–20% in elderly people^10,11^. OH have been explained by a decline of venous return associated with muscle weakness, the decrease in fluid volume, the increase of venous compliance, dysfunction of the baroreceptor and the degradation of cardiac performance^12^. Orthostatic hypotension is common in those who are age 65 and older. Special cells near heart and neck arteries that regulate blood pressure can slow as aging. It also may be harder for an aging heart to speed up and compensate for drops in blood pressure. The otolith organs are vulnerable to degradation due to aging, and the vestibulocardiovascular response may be diminished with age^13^.

In our study, the prevalence of OH among all BPPV patients was 8.3%, similar to that of other studies^10^. In this study, age also had a statistically significant effect on the occurrence of orthostatic hypotension (Table 1). However, hypertension or diabetes, acting as risk factors for OH, did not have any significant effect on the occurrence of OH in BPPV patients in this study. In addition, it was found that drug-taking had a significant effect on the occurrence of OH (Table 1). Of the 20 patients with OH, five were on medication. Two had heart disease, 1 had arrhythmia, 1 took methimazole for hyperthyroidism and 1 took prazosin hydrochloride for prostate hypertrophy. Of the seven patients who took antidepressant drugs, 5 took escitalopram and 2 took paroxetine. All seven patients did not develop OH. Of the three patients who took sleeping pills, 2 took alprazolam and 1 took Triazolam. All three patients did not develop OH.

On reviewing the previous studies on OH and BPPV, it can be seen that all the studies were conducted with patients with OH and post BPPV. In a study conducted by Jeon et al.^11^, 10 (9.8%) patients out of 102 patients had OH, and the prevalence was equal to the general prevalence. The findings of the foregoing study indicated that orthostatic dizziness symptoms were not related to OH in BPPV patients, that BPPV was the most common cause of orthostatic dizziness symptoms, and that the ratio of OH was the highest among orthostatic intolerance patients. Jeon et al. also assumed that impairment of VSR in BPPV was a cause of OH.

In a study conducted by Kim et al.^8^, when post-BPPV patients were studied with a group with vertigo symptoms and a group without vertigo symptoms, 11 out of 58 patients (19%) were OH patients, and the group with vertigo symptoms had significantly more OH patients. Interestingly, the foregoing study showed a higher ratio of OH compared to other studies because it used continuous BP monitoring. Kim et al. explained that continuous BP monitoring is a more sensitive test compared to BP monitoring at 1-min intervals because it can observe brief episodes of transient OH.

In the study conducted by Kim et al., the medical history and drug-taking of patients with OH were investigated, and contrary to our study, it was concluded that the possibility for drugs or medical history to cause OH in BPPV patients was very low.

In a study conducted by Pezzoli et al.^9^, the OH co-occurrence rate of patients with residual dizziness after BPPV treatment was 34%, which was significantly higher than that of the general population. However, the presence or absence of OH was not statistically related to age, gender, or symptoms of dizziness, and the number of samples was not large. The study conducted by Pezzoli also speculated that OH occurred because abnormalities in the otolith organs affected the control of the sympathetic nerves including the VSR.

In a study conducted with 248 patients with vertigo under the age of 65 years, Aoki et al.^14^ reported that the absent vestibular evoked myogenic potential group had a significantly higher ratio of OH and that this supported the hypothesis that otolith organ dysfunction induces OH. However, BPPV does not immediately mean the dysfunction of the otolith organ, and if the dysfunction due to the degeneration of the otolith organ causes OH, there may be an indirect relationship between otolith organ dysfunction and OH but it is not clear to what extent the otolith organ dysfunction affects the occurrence of OH 1 min after standing up in the HUTT test because the blood pressure drop due to VSR disorder affects the occurrence of OH within several seconds after standing up, earlier than the baroreceptor.

This study began with the following questions. A hypothesis that the importance of metabolic diseases such as hypertension and diabetes as risk factors for BPPV are growing and since these risk factors commonly affect the occurrence of OH, there may be some relationships between OH and BPPV.

The hypothesis of other studies that OH may occur temporarily due to the dysfunction of the otolith organ partially agrees that BPPV may act as a risk factor for the occurrence of OH, but since the annual prevalence of BPPV is around 1%^13^, the prevalence of OH is significantly higher than that of BPPV so that the hypothesis that OH affects the otolith organ and can affect the occurrence or recurrence of otoliths was considered more reasonable.

Therefore, contrary to the existing hypothesis, a hypothesis that OH may affect BPPV was established to conduct this study.

In this study, age and drug-taking were observed to be statistically significant as factors affecting the occurrence of OH in the BPPV patient group using logistic regression analysis. Although both factors were previously known as risk factors for OH, this study can be said to be significant in that it showed the same result in BPPV patients too. The age variable used in this study is a confounding variable that is causally related to the occurrence and recurrence of BPPV and OH, and it also has the nature of an effect modifier. An effect modifier refers to a confounding variable that causes different degrees of disturbance depending on its layers. Also drug-taking may be a confounding variable. Age and drug-taking can be said to act as confounding variables and as effect modifiers.

In a comparison of the three groups using polychotomous logistic regression analysis (Table 3), the risk of recurrence was significantly lowered in the no recurrence group compared to the multiple recurrence group when there was no OH. This is the first study supporting the hypothesis that OH may affect BPPV recurrence. There was no statistical significance of the association of OH between the no recurrence group and the single recurrence group, and it was not statistically significant enough to view OH as a factor affecting the recurrence of BPPV in general. However, unlike previous studies, this study is meaningful because it is not a study on the effect of OH on the vertigo in post-BPPV patients but is a study that shows the fact that OH present at the time of diagnosis of BPPV may act as a factor affecting the recurrence later.

In addition, the effect of drugs on the recurrence of BPPV is a newly discovered area. To this author’s knowledge, few studies have mentioned the effect of drugs as a factor for recurrence of BPPV. The study findings indicating that the risk of recurrence was reduced between the no recurrence group and multiple recurrence group when drugs such as heart disease drugs, depression drugs, sleeping pills, thyroid drugs, and prostate drugs investigated in this study were not taken.

There are studies on spontaneous intracranial hypotension (SIH) that can explain the mechanism by which OH affects the otolith organs. It is well known that SIH causes audiovestibular impairments^6^.

In a study conducted by Choi et al., 62% of SIH patients showed audiovestibular impairment, and SIH patients showed peripheral type nystagmus, which was thought to have been caused by endolymphatic hydrops and irritation of the vestibulocochlear nerve.

In a study by Xia et al., too, venous engorgement in the brain was identified as a factor for the development of BPPV in patients with SIH, and low cerebrospinal fluid (CSF) pressure was also associated with the development of BPPV^15^. When patients experience SIH, the decreased CSF pressure is directly transmitted to the perilymph, resulting in compensatory endolymphatic hydrops mimicking Menière’s disease. Then hydropically induced damage to the maculae of the utricle and saccule may predispose patients to BPPV^7,16^.

There are two possible mechanisms which can be thought to be the mechanisms through which OH causes BPPV.

First, the reduction of the perilymph due to CSF hypovolemia would result in compensatory endolymphatic hydrops that will in turn generate vertigo and auditory dysfunction.

Second, there is an assumption that the irritation of the vestibular and cochlear nerves that are present in the internal acoustic canal due to the venous engorgement that occurred in the internal acoustic canal affects the otolith organ.

The venous engorgement of the internal acoustic canal has been identified in magnetic resonance imaging studies^17^. As such, it is speculated that the hypofunction or dysfunction of the otolith organ according to the two mechanisms that can be caused by OH may act as a trigger or risk factor for BPPV. In addition, the aspect that orthostatic headache persists for several months after treatment of SIH patients was speculated as indicating that the recovery from the imbalance between CSF and endolymph takes quite a long time^17^. Therefore, it is assumed that in the case of OH found when BPPV occurred, the momentary decrease in perfusion due to the hypovolemic state may lead to an unexpectedly long time for the recovery of the otolith organ and may affect the recurrence later.

For similar reasons, BPPV patients with cardiovascular disease such as hypertension show a low ratio of first feeling postural vertigo and take longer to diagnosis^18^. For this reason, they often feel vertigo similar to BPPV symptoms, and the threshold for feeling dizziness is lowered so that it is difficult for them to feel symptoms such as vertigo. The hypofunction of the otolith organ due to the influence of OH, etc. is thought to make the patient unable to detect vertigo due to positional changes well, and the old BPPV in turn affects the sympathetic nervous system, which in turn creates a vicious cycle that adversely affects the otolith organ. There is evidence from studies conducted on animals and humans that vestibular lesions result in a diminished VSR and consequently in an increased intolerance to the orthostatic position^9^.

In this study, HUTT, a passive standing method, was measured at 1-min intervals. There are two methods of tests for measuring OH: the active standing test and the passive standing test. The two methods may have different blood pressure responses. Most studies have used the active standing test, but many elderly people cannot easily convert from the lying position to the standing position. Therefore, it is difficult to accurately measure changes in blood pressure during active standing test, especially in the elderly, and HUTT is considered to be an easier test through which it is easier to detect OH^19^.

In the case of active standing, the lower extremity muscles are used to sufficiently stimulate the sympathetic nervous system thereby inducing vestibular sympathetic reflexes, but the foregoing is not the case in the case of passive standing so that the drop in blood pressure may be smaller^14^. Therefore, the OH found during HUTT reflects the abnormalities of the overall autonomic nervous system rather than the function of the otolith organ.

HUTT does not reflect all patients' vertigo, because the blood pressure measurements at intervals of 1 min during HUTT cannot measure all of the VSR or baroreceptor activities that occur within a few seconds. Vestibular influences on the cardiovascular system operate within one heartbeat after the onset of abrupt head acceleration and VSRs may operate at a latency of 0.4 s^20^. Therefore, the blood pressure fall that meets the OH standard in the HUTT measurements at intervals of 1 min is not completely explained by the abnormality of the vestibular sympathetic nervous system reflex due to the dysfunction of the otolith organ. This is because, in general, blood pressure response due to vestibular dysfunction occurs within 30 s^14^.

This study is the first study that studied the effect of OH on the recurrence of BPPV and showed the possibility that OH could partially influence the recurrence of BPPV. The conclusions of this study about the relationship between OH and BPPV means that the absence of OH is related to the absence of recurrences.

It was difficult to statistically organize common factors that exist between OH and BPPV. If the number of samples in the multiple recurrence group was large, it is thought that the effects of the variables could be known more clearly. Advanced studies with more samples are thought to be necessary.

## Supplementary Information

Supplementary Tables.

## Data Availability

Data are available from the corresponding author upon reasonable request.

## References

[1] Rhim GI (2019). Serum vitamin D and long-term outcomes of benign paroxysmal positional vertigo. Clin. Exp. Otorhinolaryngol..

[2] Lan Y (2015). Analysis of the baroreceptor and vestibular receptor inputs in the rostral ventrolateral medulla following hypotension in conscious rats. Korean J. Physiol. Pharmacol..

[3] Jin GS, Li XL, Jin YZ, Kim MS, Park MR (2018). Role of peripheral vestibular receptors in the control of blood pressure following hypotension. Korean J. Physiol. Pharmacol..

[4] Kim HA (2019). Hemodynamic orthostatic dizziness/vertigo: Diagnostic criteria, consensus document of the committee for the classification of vestibular disorders of the Bárány society. J. Vest Res..

[5] The Consensus Committee of the American Autonomic Society and the American Academy of Neurology (1996). Consensus statement on the definition of orthostatic hypotension, pure autonomic failure, and multiple system atrophy. Neurology.

[6] Choi JH (2015). Audiovestibular impairments associated with intracranial hypotension. J. Neurol. Sci..

[7] Xia P, Zhang SR, Zhou ZJ, Shao YQ, Hu XY (2018). Benign paroxysmal positional vertigo in spontaneous intracranial hypotension. Neurol. Res..

[8] Kim HA, Lee H (2014). Autonomic dysfunction as a possible cause of residual dizziness after successful treatment in benign paroxysmal positional vertigo. Clin. Neurophysiol..

[9] Pezzoli M (2010). Benign paroxysmal positional vertigo and orthostatic hypotension. Clin. Auton. Res..

[10] Wu JS, Yang YC, Lu FH, Wu CH, Chang CJ (2008). Population based study on the prevalence and correlates of orthostatic hypotension/hypertension and orthostatic dizziness. Hypertens. Res..

[11] Jeon EJ (2013). Clinical significance of orthostatic dizziness in the diagnosis of benign paroxysmal positional vertigo and orthostatic intolerance. Am. J. Otolaryngol. Head Neck Med. Surg..

[12] Rutan GH (1992). Orthostatic hypotension in older adults—The cardiovascular health study. Hypertension.

[13] Tanaka K, Abe C, Awazu C, Morita H (2009). Vestibular system plays a significant role in arterial pressure control during head-up tilt in young subjects. Auton. Neurosci..

[14] Aoki M, Sakaida Y, Tanaka K, Mizuta T, Ito M (2012). Evidence for vestibular dysfunction in orthostatic hypotension. Exp. Brain. Res..

[15] von Brevern M (2007). Epidemiology of benign paroxysmal positional vertigo: A population based study. J. Neurol. Neurosurg. Psychiatry..

[16] Gross EM, Ress BD, Viirre ES, Nelson JR, Harris JP (2000). Intractable benign paroxysmal positional vertigo in patients with Meniere's disease. Laryngoscope..

[17] Isildak H, Albayram S, Isildak H (2010). Spontaneous intracranial hypotension syndrome accompanied by bilateral hearing loss and venous engorgement in the internal acoustic canal and positional change of audiography. J. Craniofac. Surg..

[18] Tan J, Deng Y, Zhang T, Wang M (2017). Clinical characteristics and treatment outcomes for benign paroxysmal positional vertigo comorbid with hypertension. Acta Otolaryngol..

[19] Aydin EA, Isik TA, Soysal P (2017). Which is preferable for orthostatic hypotension diagnosis in older adults: Active standing test or head-up tilt table test?. Clin. Interv. Aging..

[20] Monahan KD, Ray CA (2002). Vestibulosympathetic reflex during orthostatic challenge in aging humans. Am. J. Physiol. Regul. Integr. Comp. Physiol..

