# Supplementary material for: Relationship between orthostatic hypotension and recurrence of benign paroxysmal positional vertigo
Source: Sci Rep. 2022 Jun 23;12:10685. doi: 10.1038/s41598-022-15029-5 (PMC9226118; doi:10.1038/s41598-022-15029-5)
Supplement: Supplementary file 1 — Supplementary Tables. [file 41598_2022_15029_MOESM1_ESM.docx]

| **Table S1.**   \| **Tests of Conditional Independence** \| \| \| \| \| --- \| --- \| --- \| --- \| \|  \| Chi-Squared \| df \| Asymp. Sig. (2-sided) \| \| Cochran's \| .510 \| 1 \| .475 \| \| Mantel-Haenszel \| .002 \| 1 \| .963 \| \| Under the conditional independence assumption, Cochran's statistic is asymptotically distributed as a 1 df chi-squared distribution, only if the number of strata is fixed, while the Mantel-Haenszel statistic is always asymptotically distributed as a 1 df chi-squared distribution. Note that the continuity correction is removed from the Mantel-Haenszel statistic when the sum of the differences between the observed and the expected is 0. \| \| \| \|   **Table S2. Polychotomous logistic regression analysis**  **Parameter Estimates** | | | | | | | | | | |
| --- | --- | --- | --- | --- | --- | --- | --- | --- | --- | --- | --- | --- | --- | --- | --- | --- | --- | --- | --- | --- | --- | --- | --- | --- | --- | --- | --- | --- | --- | --- |
| recurrence | | B | Std. Error | Wald | df | Sig. | Exp(B) | 95% Confidence Interval for Exp(B) | | |
|  |  |  |  |  |  |  |  | Lower Bound | Upper Bound | |
| no | Intercept | 50.064 | 2575.275 | .000 | 1 | .984 |  |  |  | |
|  | [OH=1] | -15.710 | 1.089 | 208.028 | 1 | .000 | 1.504E-7 | 1.779E-8 | 1.272E-6 | |
|  | [OH=2] | 0^b^ | . | . | 0 | . | . | . | . | |
|  | [Hypertension=1] | .180 | .990 | .033 | 1 | .856 | 1.197 | .172 | 8.339 | |
|  | [Hypertension =2] | 0^b^ | . | . | 0 | . | . | . | . | |
|  | [Diabetics=1] | -16.164 | 2575.275 | .000 | 1 | .995 | 9.549E-8 | .000 | .^c^ | |
|  | [Diabetics =2] | 0^b^ | . | . | 0 | . | . | . | . | |
|  | [Drug=1] | -15.750 | .646 | 594.644 | 1 | .000 | 1.445E-7 | 4.073E-8 | 5.123E-7 | |
|  | [Drug =2] | 0^b^ | . | . | 0 | . | . | . | . | |
|  | [Sex=1] | 1.149 | 1.095 | 1.101 | 1 | .294 | 3.156 | .369 | 27.001 | |
|  | [Sex=2] | 0^b^ | . | . | 0 | . | . | . | . | |
|  | [age= 1.00] | .182 | .884 | .042 | 1 | .837 | 1.199 | .212 | 6.782 | |
|  | [age= 2.00] | 0^b^ | . | . | 0 | . | . | . | . | |
| single | Intercept | 48.760 | 2575.274 | .000 | 1 | .985 |  |  |  | |
|  | [OH=1] | -14.868 | .000 | . | 1 | . | 3.489E-7 | 3.489E-7 | 3.489E-7 | |
|  | [OH=2] | 0^b^ | . | . | 0 | . | . | . | . | |
|  | [Hypertension =1] | .121 | 1.173 | .011 | 1 | .918 | 1.129 | .113 | 11.239 | |
|  | [Hypertension =2] | 0^b^ | . | . | 0 | . | . | . | . | |
|  | [Diabetics =1] | -16.813 | 2575.275 | .000 | 1 | .995 | 4.989E-8 | .000 | .^c^ | |
|  | [Diabetics =2] | 0^b^ | . | . | 0 | . | . | . | . | |
|  | [Drug=1] | -16.721 | .000 | . | 1 | . | 5.475E-8 | 5.475E-8 | 5.475E-8 | |
|  | [Drug=2] | 0^b^ | . | . | 0 | . | . | . | . | |
|  | [Sex=1] | -15.357 | 1210.064 | .000 | 1 | .990 | 2.140E-7 | .000 | .^c^ | |
|  | [Sex=2] | 0^b^ | . | . | 0 | . | . | . | . | |
|  | [Age= 1.00] | .264 | 1.027 | .066 | 1 | .797 | 1.302 | .174 | 9.741 | |
|  | [Age= 2.00] | 0^b^ | . | . | 0 | . | . | . | . | |
| a. The reference category is: multiple recurrence.  b. This parameter is set to zero because it is redundant.  c. Floating point overflow occurred while computing this statistic. Its value is therefore set to system missing. | | | | | | | | | | |
|  | | | | | | | | | | |
|  | | | | | | | | | |  |
